# How labeling of genetically modified foods affects consumers’ purchase intentions: a multi-contextual analysis

**DOI:** 10.1080/21645698.2025.2572191

**Published:** 2025-10-08

**Authors:** Zheng Yang, Yingdi Jiang, Yun Feng, Guoyan Wang

**Affiliations:** The School of Communication, Soochow University, Suzhou, China

**Keywords:** Dunning-Kruger effect, GM labeling, purchase intention, risk perception, S-O-R model

## Abstract

As a critical carrier for ensuring consumer right-to-know and facilitating risk communication, the effectiveness of genetically modified (GM) labeling is influenced by cognitive biases, yet its behavioral impact remains underexplored, particularly in non-Western contexts. Through a dual-context online experiment (edible soybean oil vs. non-edible cotton, *n* = 800) conducted in China, this study examines how GM labeling affects purchase intentions, incorporating the roles of risk perception and moderating effect of metacognitive bias. The results reveal that risk perception mediates this relationship, while metacognitive ability significantly moderates it. More importantly, the results indicate that the audiences with different cognitive characteristics also have diverse cognitive effects and psychological pathways toward similar GM labels. These findings surpass traditional “knowledge-attitude” linear paradigms and further offer practical insights for policymakers: tailored GM labeling regulations and segmented communication strategies should be developed based on cognitive typologies to improve consumer understanding and decision-making.

## 1. Introduction

As a typical example of modern agricultural biotechnology, the industrialization process of genetically modified (GM) technology has always been accompanied by a complex mix of technical effectiveness and public perception.^1^ From the perspective of technology application, ISAAA data shows that, as of 2019, the global planting area of GM crops covered 29 countries, totaling 189.8 million hectares, and involved the enhancement of insect resistance and herbicide tolerance traits in major food crops such as soybeans and corn, significantly enhancing agricultural production efficiency.^2^ The international scientific community generally believes that GM crops are a key technological way of addressing the challenges of global population growth and food security.^3^ Some studies specifically identify GM crops as the core solutions to address food security.^4,5^ However, as Qiam and Kouser (2013) note, the role of GM crops for food security is also the subject of public controversy, where there is a sharp contrast between the rapid advancement of technology application and slow public acceptance around GM crops. Taking the Chinese market as an example, although the Chinese government has vigorously promoted the industrialization of GM crops, the rate of consumer opposition to GM foods still rose from 11% in 2002 to 41.4% in 2016,^6^ with 54.45% of consumers unwilling to buy GM foods.^7^ This binary divergence between “high acceptance by producers” and “high scepticism by consumers” reveals a deep gap between scientific consensus and social perception. Thus, the public’s negative attitude has been found to significantly delay the industrialization of GM technology.^2^ From the perspective of the market transmission mechanism, insufficient consumer acceptance will suppress the vitality of demand and prompt biotech companies to shift their research and development resources to non-GM areas,^8^ suggesting that public attitude has a profound impact on the development of the GM industry.

Public attitudes toward GM crops and foods have been found to be significantly influenced by its commercial information representation, especially the product labels.^2^ Currently, to safeguard consumers’ right to know and choose, the international community generally requires labeling of GM foods, but there are significant differences among countries in terms of labeling thresholds, coerciveness, and content.^9^ Studies have shown that GM labeling can induce both positive and negative consumption effects simultaneously. For instance, consumers expect clear labeling on GM crops,^10^ and clearly labeling something as GM can also to some extent reduce consumers’ panic about GM foods^11^ But more studies highlight the potential negative effects of GM labeling, especially triggering negative perceptions among consumers, such as products with GM labeling often being regarded as inferior,^12^ significantly reducing consumers’ willingness to purchase them.^13^

Thanks to varied policy aims, labeling differences lead to significant market differentiation: the market share of GM foods dropped significantly after the EU implemented mandatory labeling,^14^ while the US maintained a higher market growth rate through the threshold exemption system of the National Bioengineered Food Disclosure Standard (2016).^15^ Such policy effects support the “institutional cognitive construction” theory that says labeling regulations reshape the public’s risk-benefit assessment paradigm through an information framework.^16^ Further research found that different labeling systems (“GM” and “non-GM”) not only sparked widespread public discussion on GM technology, but also profoundly influenced consumer preferences and behavioral choices,^2^ confirming at the practical level that GM labeling is a key variable that influences public perceptions.

While existing studies have confirmed that GM labeling has a significant impact on public attitudes,^10,11,17,18^ its internal psychological mechanisms have not been fully explored, especially in terms of the differentiated responses of group heterogeneity. Existing literature often regards the public as a homogeneous group and finds that consumers have a tendency to distrust GM products.^19,20^ But, as a highly controversial field, public perceptions of GM technology continue to be complex and influenced by various factors,^21^ and there is a significant difference between the consumers’ subjective and objective perceptions of GM biotechnology or food.^22^ Although some studies have noted that variables such as educational attainment, cultural background, gender, and familiarity with technology may cause differences in attitude,^23,24^ such analyses are mostly confined to the context of Western developed countries and pay less attention to consumer psychology in Eastern cultures.^25^ Meanwhile, in addition to a conspicuous scarcity of focus on non-Western contexts, existing research also lacks in-depth exploration of the internal psychological mechanisms underlying consumer responses to GM labeling – particularly the heterogeneous psychological mechanisms across different demographic groups. In light of these research gaps, deconstructing the group- and product-specific psychological dynamics in GM labeling, especially within non-Western context, is necessary.

Based on the research gap above, this study focuses on the following question: through what psychological mechanism does GM labeling affect consumers’ willingness to purchase and how do the effect pathways vary between different groups and products in China, and a series of more detailed hypotheses, such as Chinese consumers’ risk perception plays a significant mediating role between GM labeling and purchase intentions. To address the above research questions and hypotheses, this study integrates the Stimulus-Organism-Response (S-O-R) framework and cognitive psychology perspectives to analyze the cognitive logic behind consumer decision-making, moving beyond the limitations of traditional technology adoption paradigms. It provides a theoretical basis for reconciling scientific consensus with social cognition and offers empirical support for differentiated labeling policies. The findings further contribute by proposing a cross-contextual theoretical model that incorporates group-level and product-related variations, helping to bridge key gaps between science and public perception. Moreover, by examining these mechanisms in an understudied non-Western setting, the study challenges the universal applicability of Western-centric findings and supports the design of culturally and cognitively attuned labeling policies.

## 2. Literature Review and Research Hypotheses

### 2.1. GM Labeling and Consumer Decision-Making

In the process of globalization, the industrialization and commercialization of GM technology have become common concerns for the international community.^4^ Some meta-analysis has shown that GM technologies can bring a series of economic and social benefits, such as reduced use of chemical pesticides, increased crop yields, and increased profits for farmers, as well as providing sustainable nutrition, especially for developing countries.^26,27^ Other systematic reviews have shown that even with such benefits, there is a highly contradictory or oppositional attitude among the public, not only in the West, but also in non-Western countries.^1,10,28,29^

Due to the large gap between scientific judgment and public opinion on GM technology, governments around the world are faced with the dilemma of how to regulate the marketing of GM foods,^30^ including GM labeling.^31^ Although countries have developed diverse labeling policy systems due to differences in historical and cultural traditions, technological development levels, and public acceptance,^31^ GM labeling – as a core system for food information disclosure – has been established through legislation in many nations.^32^ This universal policy practice stems from the crucial role of labeling on public perceptions: empirical research shows that labeling significantly affects consumer decision-making by alleviating information asymmetry, and that consumer acceptance drops sharply when products are labeled as GM.^33^ International comparisons of policy effects further confirm this mechanism: the market share of GM foods declined after the EU implemented a mandatory labeling regime with a 0.9% threshold,^14^ while the US maintained a relatively good annual consumption growth rate with a voluntary labeling regime.^15^ Evidence suggests that commercial information, including labels, represents a key information cue, revealing and helping to meet consumers’ preferences for GM products,^1,33^ and further influencing public perception, acceptance and willingness to consume GM products.^10,18^ Some studies have also found that such “influence,” especially potential negative “influence,” can be offset to some extent. For instance, one study found that advertising GM products with expert opinions can significantly reduce consumers’ perceptions of risk and further raise their awareness and acceptance toward GM products.^34^

Focusing on the labeling, the mechanism of commercial GM information is rooted in their information framing effect and technical visibility characteristics, which further profoundly influences consumers’ attitudes and willingness to consume GM products.^34,35^ This is especially true in China, where the public’s attitudes toward GM products are complex.^18,35^ According to a study by Zhao and colleagues (2019), nearly 80% of Chinese consumers accept food labeled as non-GM, 57% accept food without any labels, and approximately 40% accept food labeled as containing GM organisms.^36^ According to information framing theory, labeling reconstructs consumers’ decision-making reference framework through “attribute highlighting.”^37^ Although some studies have shown that labels only provide objective information rather than affective cues^38^ with limited signaling effects and do not have a significant impact on consumers’ attitudes and shopping behavior biases,^39^ other research suggests that GM labels can amplify consumers’ preexisting shopping tendencies.^10^ Many studies have found that consumers rate non-GM foods higher than GM foods^40^ and are willing to pay extra for non-GM foods.^41^

Through studies of potatoes, crisps, milk, chocolate, corn, and cornmeal wafers, scholars have found that if consumers were able to choose between GM and GM foods, there was a potential market for GM foods in the US.^42^ When products are labeled as “containing GM ingredients,” their technical attributes are forced to be visible.^35^ While GM crops are rapidly accepted for cultivation by farmers in many countries and in the global food and feed markets, consumers’ acceptance is often limited.^43^ GM labeling has been found to be significantly positively correlated with a decline in consumers’ willingness to pay for such products.^40^ On the other hand, “non-GM” labeling forms a positive framing effect.^44^ Studies have shown that non-GM labeling can serve as a “safety cue” to reduce consumers’ cognitive concerns about related products, and further generate a reverse incentive effect, whereby “non-GM” labeling constructs a cognitive shortcut of “nature = safety” and increases consumers’ willingness to purchase.^44^ One study also highlighted that the mandatory labeling of genetically engineered food could effectively reduce the US consumers’ opposition to it.^45^ Empirical data also shows that labeling something as “non-GM” can increase the premium of products, especially among organic food consumers.^41^ Some studies further show that absence-claim labeling (non-GM) does not have a negative impact on the demand for related conventional products, but consumer demand for unlabeled products is significantly enhanced with the introduction of presence-claimed GM labels.^46^ Thus, the impact of GM labels on consumers is complex and diverse. But whether the effect is positive or negative, direct or indirect, the significant impact of GM labeling (including non-GM) on consumers has reached a consensus in the academic community.^10,18^

The above effects of GM labeling and policies are highly influenced by cultural environment.^47,48^ Many studies have focused on the Western cultural context that emphasizes personal consumerism,^41,44^ with less research on the more naturalistic Chinese context. In the context of the ongoing optimization of China’s GM labeling policy, the impact of GM labeling on purchase intention has not been fully explored. The dual effect of “GM” and “non-GM” labeling is essentially an institutionalized choice of the information framework: the former builds a safety narrative through “negative labeling,”^16^ while the latter reinforces risk warnings through “affirmative labelling.”^14^ Chinese consumers, in the context of “GM stigmatization” and “GM non-naturalization,” tend to seek psychological compensation through “non-GM” labels.^9^

To summarize, we cannot fully assert whether GM labels will have a positive or negative effect on consumers. Thus, a more general research question is first proposed: does GM labeling have an impact on consumers’ purchase intentions? To investigate, this paper uses signal theory to operationally argue that the “non-GM” labeling may increase purchase intention through “safety cues,”^41^ while the “GM” labeling may reduce consumers’ purchase intentions. Thus, the first research hypothesis of this paper is proposed:

H1: GM labeling negatively predicts the purchase intentions of Chinese consumers.

### 2.2. The Mediating Mechanism of Risk Perception

Consumer behavior studies in recent years have revealed that the impact of GM labels on purchase decisions is not simply linear but works through complex and diverse psychological mediating mechanisms.^40^ Many empirical studies have shown that labeling information involves mediating processes such as cognitive assessment and emotional regulation before it ultimately influences consumer behavior,^49^ such as amplifying consumers’ fears.^50,51^ This mechanism could theoretically be systematically explained by the Stimulus-Organism-Response (S-O-R) paradigm, which holds that external stimuli trigger changes in an individual’s mental state, that in turn leads to behavioral responses.^52^ Whether through exploration of the attribution of the public’s overall attitude toward science and technology or of the factors influencing the public’s attitude toward GM technology, previous scholars have identified cognitive and psychological-level variables such as scientific knowledge, risk perception, benefit perception, and values.^53^

Among the many variables that may be affected by GM labeling, such as price, knowledge and trust in science,^17^ risk perception is widely seen as a key mediating variable linking identification stimulus to purchase decisions.^1^ Protection Motivation Theory (PMT) further dissects this mechanism: consumers form a risk perception matrix by assessing the severity of the threat and their own vulnerability, which in turn drives their avoidance behavior.^54^ For example, the “pesticide-free” label significantly boosts willingness to purchase organic food by weakening the perception of chemical risks.^41^ Focusing on GM products, the study found that public attitudes toward their associated risks significantly affected the willingness of consumers in the US and China to purchase GM foods.^55^ The uncertainty inherent in GM foods has also been found to further amplify consumers’ concerns about their risks.^56^ Some studies have also shown that when consuming GM products, consumers’ perceived benefits are a more influential factor than perceived risks,^57^ especially in the Chinese context;^58^ moreover, GM labeling does not necessarily directly lead to consumer identification with the product.^36^ Most studies indicate that GM labeling will ultimately affect consumers’ purchasing decisions by amplifying their risk perception.^17,18^ And, as discussed above, such risk perception of GM products may be offset by including expert knowledge in associated commercial information.^59^

Further, how GM labels are presented can directly influence consumers’ perceptions of their risks.^32^ According to the Framing Effect theory, the presentation of information can significantly alter an individual’s perception of risk.^60^ For example, negative labels such as “non-GM” can amplify consumers’ perceptions of the threat posed by GM technology by emphasizing “risk-averse” narratives.^32^ Positive labels, such as “contains GM ingredients,” may cause higher uncertainty anxiety by directly exposing the technical attributes of the product.^61^ Moreover, risk perception is regarded as a decisive factor in the public’s attitude toward GM technologies and their products,^62^ and studies have found that stronger risk perception is less conducive to the formation of a positive public attitude toward GM.^63^

Thus, GM labeling is not only a carrier of information but also a “stimulus source” that shapes the public’s perception of risk. Research shows that consumers’ concerns about the health and ecological risks associated with GM products can explain reduced willingness to pay for them.^28,49^ These findings confirm the pivotal position of risk perception in the “stimulus-response” chain. However, most studies above are focusing on the Western context, in the Chinese context where the public’s perception of risk is vastly different from that of the West,^49^ the mechanism by which GM labeling influences consumers’ willingness to purchase through risk perception remains unclear. Therefore, we raise a relatively open question: does GM labeling really trigger consumers’ risk perception in the Chinese context? According to Hofstede’s model, in Chinese society, which has a strong culture of aversion, the public has a more pronounced tendency to avoid uncertainty and risk.^64^ But it remains to be seen whether this cultural trait is further reflected in the relationship between GM labeling and consumption. Therefore, based on the S-O-R model, this paper assumes that in the context of Chinese culture, risk perception plays a key transmitting role between GM labeling and purchasing behavior. We further operationally propose the following hypotheses based on the research question above:

H2a: GM labeling can positively predict the risk perception of Chinese consumers.

H2b: The risk perception of Chinese consumers negatively predicts their willingness to purchase.

H2c: Chinese consumers’ risk perception plays a significant mediating role between GM labeling and purchase intentions.

### 2.3. The Dunning-Kruger Effect: A Moderating Mechanism of Metacognitive Bias

GM purchase decision-making is not only about objective knowledge reserves, but also metacognitive ability – an individual’s monitoring and evaluation of their own cognitive levels.^65^ The impact of GM labeling on individual risk and decision-making has also been found to be regulated by both cognitive ability and knowledge level.^66^ It is notable that knowledge level itself has a dual dimension – objective knowledge reserves and subjective knowledge evaluations – and the difference between the two has been proven to significantly influence the public’s cognitive patterns and behavioral tendencies toward specific technologies.^67^ There are significant differences in the acceptance of GM labels among different consumer groups: the same label may not have a consistent effect on all populations.^68^ Therefore, the “knowledge-attitude” paradox and its effects on different consumers’ behaviors has drawn much academic attention in the study of public perceptions of GM technology. Cross-national comparative studies have shown that there is no stable positive correlation between the public’s level of biotechnology knowledge and their attitude toward GM products.^69^ It is notable that although highly educated groups generally agree with the scientific community’s consensus on the safety of GM products, their sensitivity to risk perception inhibits the efficiency of converting technological identification into consumer behavior.^70^ These findings pose a challenge to the explanatory power of the traditional knowledge deficit model, suggesting that cognitive science research requires a perspective shift: under the condition that knowledge accumulation is insufficient to explain attitude formation, the research focus should be shifted to the deconstruction analysis of cognitive schemas, including but not limited to cognitive frameworks, risk assessment models, and value ranking mechanisms in the individual’s information processing method. From this it can be inferred that the root cause of the differences in consumer decision-making lies not only in the disparity of objective knowledge levels, but may also be associated with metacognitive bias, which may further intensify the heterogeneous impact of GM labeling on purchase intentions.

In analyses of heterogeneous cognitive populations, due to its dual role in explaining cognitive bias patterns, the Dunning-Kruger effect has been widely employed to account for systematic misjudgments in self-assessment across diverse groups.^65,71^ The Dunning-Kruger effect reveals a paradox: low-cognitive groups tend to overestimate their judgment and fall into the decision-making trap of “the ignorant are fearless.”^71^ In the face of specific scientific issues, numerous studies have found that the public undergoes a distinct Dunning-Kruger effect, i.e. they tend to be unable to accurately assess and even overestimate their objective knowledge level, which further affects their attitude and perception toward specific technologies. For example, Motta (2018) found that the extreme stance of the anti-vaccine community is significantly associated with their knowledge deficit and inflated self-assessment;^67^ Krawcayk et al. (2013) also pointed out that the direction of perceived knowledge levels may vary significantly across different scientific issues.^72^ Research focused on the issue of GM foods shows that extreme attitudes against GM foods among the American public are increasing, while objective knowledge related to science and genes is decreasing, and self-perceived understanding of GM foods is on the rise.^65^ This mismatch between knowledge levels and self-assessment may reshape the formation mechanism of risk perception: consumers who overestimate their own knowledge levels tend to ignore risk warnings and rely more on intuitive decisions;^65^ in contrast, groups that underestimate their own knowledge levels exhibit more systematic patterns of information processing, with significantly reduced emotional responses in their decision-making.^73^

Risk perception has also been found to be closely associated with an individual’s level of knowledge.^74^ For example, studies have found that the public’s level of knowledge about the disease profoundly affected their perception of the risk associated with the Covid-19 pandemic.^74^ However, these studies focused on the audience’s objective perception of knowledge or relied solely on the audience’s subjective evaluation of knowledge. Few studies have evaluated the regulatory effect of knowledge level on risk perception by considering the difference between the two (the Dunning-Kruger effect^75^ – the role of cognitive bias in the path between GM labeling and purchasing behavior is not clear, thus further investigation is needed. In response to the issue of GM labeling and consumption in this article, it is speculated that the Dunning-Kruger effect may influence consumer behavior through multiple pathways. This theoretical framework breaks through the single-dimensional analysis model of traditional research and provides a new research path for revealing the complex mechanisms of GM decision-making, leading to the following research question:

**RQ**: Dunning-Kruger effect groups (underestimating/correctly assessing/overestimating) exhibit differential pathway effects between exposure to GM labeling and risk perception.

**Figure 1.**
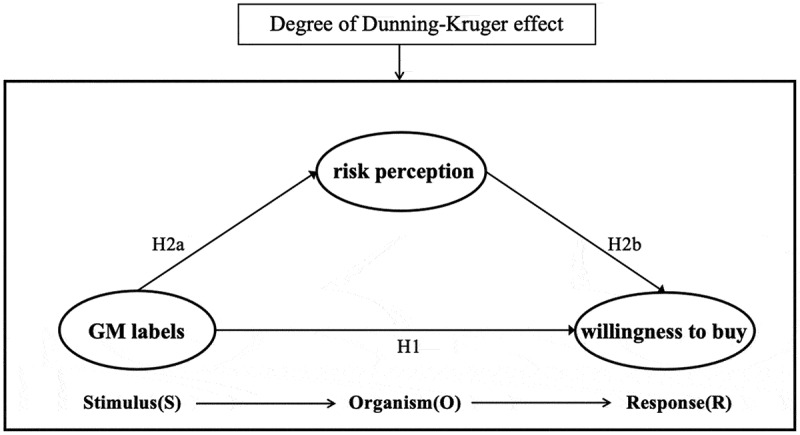
Consumer purchase intention mechanism model and research hypotheses based on the S-O-R theoretical model.

Based on the above research questions and hypotheses, the following model of the potential impact mechanism of GM labeling on consumer purchase intention was created (Figure 1).

## 3. Research Methods

### 3.1. Sampling and Subjects

To answer the above research questions, an online experiment was conducted in December 2024, with participants recruited through compensated quota sampling via an online platform (www.credamo.com.), which has been validated to have a good data collection effect,^76^ with quotas set for regional representation, age, and gender to ensure demographic diversity. The experimental method is believed to effectively explore general patterns across contexts and has been widely applied in the study of the impact mechanism of specific information clues (such as labels) on consumer attitudes and behaviors.^77^

Studies show that there are significant differences in consumer responses to commercial information regarding different types of GM products.^51,78^ Therefore, the experiment was conducted in two distinct scenarios: Scenario 1 featuring “Soybean oil” and Scenario 2 featuring “Cotton.” After screening for attention and response duration, a total of 800 valid questionnaires were collected (400 for each Scenario). This study used G * Power 3.1 software for preliminary calculation of sample size. According to Cohen’s (1988), based on f = 0.25 (moderate effect), α = 0.05、1-β = 0.80, the calculation results show that the minimum required sample size is 196. A total of 800 valid samples was collected in the actual experiment, indicating sufficient sample size and good statistical testing ability.

In the two experiments, the proportion of women was 67.0% (*n* = 268) in Scenario 1 and 70.3% (*n* = 281) in Scenario 2; the age range of participants was 18 to 65 years old (*M* = 30.77; SD = 8.569) and 17 to 66 years old (*M* = 31.01; SD = 7.064) respectively. The highest proportion of educational attainment among the subjects was undergraduate (Scenario 1: 69.3%, *n* = 277; Scenario 2: 67.0%, *n* = 286). In terms of the frequency of purchases of consumer goods, a seven-point Likert scale was used (1 = never, 7 = frequent). The scores for the two scenarios were: Scenario 1: *M* = 5.88, SD = 1.004 and Scenario 2: *M* = 5.78, SD = 1.021. Critically, demographic variables (e.g., age, gender, income) exhibited no statistically significant differences between the two experimental groups (*p* > .05 for all comparisons), ensuring baseline equivalence in population characteristics.

### 3.2. Experimental Procedure and Materials

As mentioned above, to enhance the external validity and universality of the research model in this study, the researcher selected two topics with significant differences in issue relevance – public awareness and degree of controversy – as experimental scenarios and conducted online experiments respectively. Firstly, these two scenarios were chosen because the GM products currently approved for import in China comprise cotton, corn, soybeans, rapeseed, sugar beet, papaya, alfalfa, and sugarcane. Among these, GM cotton and soybeans are the types most commonly encountered by consumers in their daily lives. Furthermore, they are involved in entirely different consumption sectors (textile raw materials and food raw materials), providing different scenarios for studying the potential effects of GM labeling. Secondly, soybean oil, as a food-related topic, has a high degree of daily influence and issue relevance. Edible oil is an indispensable part of the daily diet of respondents, and related food safety incidents have frequently emerged in public,^79^ generating a high foundation of public awareness and motivation for information participation. In the context of the “GM” issue, edible oil products, which are directly related to the safety of eating, are often more likely to trigger perceived risks and emotional responses among the public, thereby triggering stronger judgments and behavioral intentions. Soybean oil thus has typical high involvement characteristics.^80^ By contrast, cotton, as a topic of daily necessity, is widely present in life, but its “GM” attribute is less well understood by the public and does not trigger immediate risk associations. The public’s consumption of cotton products usually does not involve immediate health consequences, and the issues it triggers are more dependent on media cues or passive acceptance, belonging to the low-involvement category, with relatively low public familiarity and emotional investment. At the same time, there is no broad social consensus on the controversy and risk perception of “GM” in the cotton sector, and there is some ambiguity and marginality.

**Figure 2.**
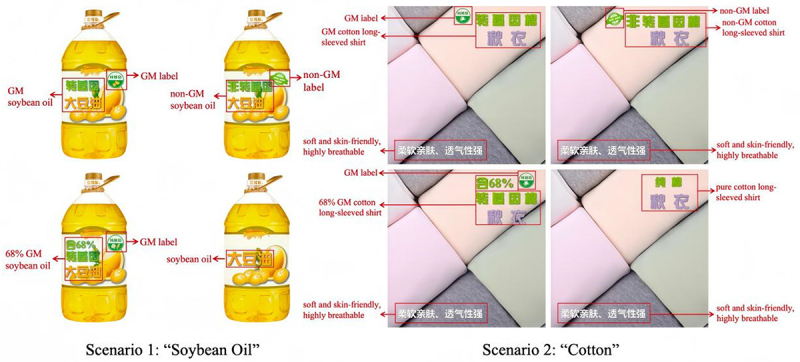
Soybean oil and cotton experiment stimulus material.

During the experiment, participants voluntarily clicked on the questionnaire link and were randomly assigned to four different experimental settings in the two scenarios. The overall process was divided into the following stages: in the first, participants answered demographic questions, assessed their familiarity with GM knowledge, and objectively measured their familiarity with it using true or false questions. In the second stage, the subjects were presented with experimental stimuli in the form of pictures. The images showed products labeled as either GM or non-GM, or without a label. In Scenario 1, the displayed products were respectively labeled “GM soybean oil,” “68% GM soybean oil,” “non-GM soybean oil,” and “soybean oil” (as shown in Figure 2). In Scenario 2, the products shown were respectively labeled “GM cotton long-sleeved shirt,” “68% GM cotton long-sleeved shirt,” “non-GM cotton long-sleeved shirt,” and “pure cotton long-sleeved shirt.” In each group, the products were the same, with the only difference being the presentation of information in the product names (as shown in Figure 2). In the third phase, participants reported their risk perceptions and desire to purchase the product in the experimental material. At the end of the experiment, the subjects were informed that the pictures they had seen were experimental materials, and the information about the composition and content of the products was fictional.

### 3.3. Study Variables and Measurement Methods

#### 3.3.1. Perceived Risk

Risk perception is an individual’s subjective assessment and interpretation of potential dangers and their likelihood, assessed through cognitive processes. In this study of GM labels, the risk perception measurements of the subjects for different labeled products referred to and adapted the scales of Dong et al. and Siegrist.^81,82^ The participants evaluated their perception of the risks associated with the displayed product using a Likert scale of seven (with 1 indicating strong disagreement and 7 strong agreement) that responded to the following statements: “I am concerned about the safety of GM products”; “I am concerned that the production of GM products will damage the ecological environment”; “I’m concerned that using GM products will affect health”; “I’m worried that the market for GM planting will be dominated by international seed giants, affecting our country’s social security”; and “I’m worried that GM oil doesn’t taste good/that GM cotton products are uncomfortable.” Overall scale data: *M* = 4.808, *SD* = 1.494, Cronbach’s Alpha = 0.916; Scenario1: *M* = 4.905, *SD* = 1.423; Cronbach’s Alpha = 0.90; Scenario2: *M* = 4.711, *SD* = 1.558, Cronbach’s Alpha = 0.929.

#### 3.3.2. The Dunning-Kruger Effect

The Dunning-Kruger effect focuses on individual biases in people’s perception of their own abilities.^83^ Following Kruger and Dunning’s 1999 methodology,^71^ the questionnaire first asked participants to self-assess their knowledge of genetically modified (GM) technology using a 7-point Likert scale, with 1 indicating “very unfamiliar” and 7 indicating “very knowledgeable.” Seven true or false questions about GM products, involving both scientific questions and policy-related questions, were then provided (see Appendix). The difference between self-rated scores and test scores was used as the basis for population division. People who scored between −1 and 1 were considered as having evaluated themselves correctly, those with less than −1 underestimated themselves, while those with more than 1 had overestimated themselves. The reliability of subjective evaluation in the two scenarios was 0.618 (soybean oil) and 0.623 (cotton), both within an acceptable range.

#### 3.3.3. Experimental Manipulation Tests

To test whether the experimental stimulus successfully manipulated participants’ perceptions of the (non-)GM labeling of different products, the study asked, “whether the labels displayed on the products are clear and understandable?” The results showed that after excluding samples that failed the attention test, all participants were influenced by the (non-)GM labeling on the product. The manipulation was successful when the match between the options of the test questions and the characteristics of the picture content watched by the subjects was 100%. To further check the results of manipulation, we examined the differences in purchase intention (core dependent variable) between different groups. It was found that, except for a very small number of groups, the differences between most groups were significant (Table 1). This indicates that not only is the stimulation of labels successful, but it also has a significant effect on consumers’ purchase intention.

**Table 1.** Differential testing of purchase intention among different experimental groups.

Variables		Mean difference	*p*
68% GM labels	GM labels	0.170	0.232
	Non-GM labels	−0.665	0.000
	Blank control	−0.205	0.149
GM labels	GM68% labels	−0.170	0.232
	Non-GM labels	−0.835	0.000
	Blank control	−0.375	0.008
Non-GM labels	GM68% labels	0.665	0.000
	GM labels	0.835	0.000
	Blank control	0.460	0.001
Blank control	GM68% labels	0.205	0.149
	GM labels	0.375	0.008
	Non-GM labels	−0.460	0.001

## 4. Data Analysis and Results

In this study, univariate ANOVA was used to examine the randomness of grouping between two experimental scenarios and within each scenario. After examining the manipulation test results, the study further analyzed the subsequent data using SPSS 26.0 and the process plug-in model 4 within it.

### 4.1. Randomization Test

To confirm randomness in terms of gender, age, education level, and procurement frequency of daily necessities between the two experimental scenarios and among the groups within each, one-way analysis of variance was used. The results showed that, first, between the two scenarios gender (*x*^*2*^(1) = 0.211, *p* = .323), age (*F*(1, 798) = 0.208, *p* = .649) and education (*F*(1, 798) = 3.504, *p* = .062) there were no significant differences, or in the frequency of purchasing daily necessities (*F*(1, 798) = 2.151, *p* = .13). In terms of Scenario 1 (*n* = 400), the results were: gender (*x*^*2*^(3) = 0.940, *p* = .237), age (*F*(3,396) = 0.640, *p* = .590), education level (*F*(3, 395) = 0.947, *p* = .418), and purchase frequency of daily necessities (*F*(3, 395) = 0.905, *p* = .439). In Scenario 2, results were: (*n* = 400), gender (*x*^*2*^(3) = 0.847, *p* = .257), age (*F*(3, 396) = 0.647, *p* = .585), education level (*F*(3, 396) = 2.647, *p* = .049), and income level (*F*(3, 396 = 0.213, *p* = .887), and there were also no significant differences in these aspects. This indicates that randomization allocation in both experiments met the requirements.

### 4.2. Results of the Experiments

#### 4.2.1. Between-Subjects Effect Test

**Figure 3.**
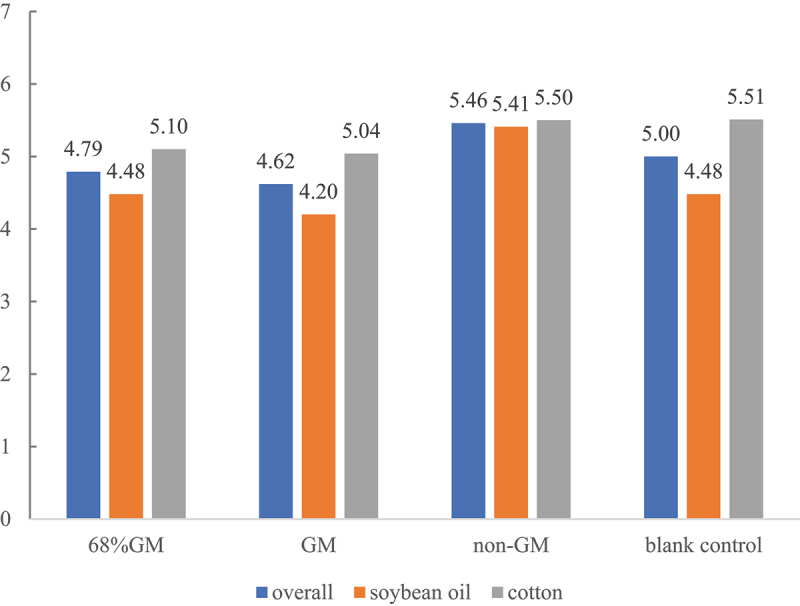
Purchase intention scores across different product groups.

To test the effects of different treatment conditions on the dependent variable, the Univariate Analysis method in the General Linear Model (GLM) was used. The test of Between-Subjects effects was used to assess whether the main and interaction effects of the independent variables on the dependent variables were significant. The study was initially validated with context (product category) as the independent variable and purchase intention as the dependent variable. The results showed that under the non-GM label condition, there was a significant difference in consumers’ willingness to purchase different categories of products (*F*(1, 396) = 19.430, *p* < .001). Purchase intention for non-GM labeled products was significantly higher than for their conventionally labeled counterparts (*F*(1, 396) = 13.110, *p* < .001). There was a significant interaction between the product category and the non-GM label (*F*(1, 396) = 13.687, *p* < .001), meaning the effect of the non-GM label varied by product category. In the food scenario, non-GM labels are thus more effective in enhancing consumers’ willingness to purchase. Consumers might be more concerned about the non-GM information of edible oil and less concerned about the non-GM label of cotton. Under the GM label condition, product category had a significant impact on purchase intention (*F*(1, 596) = 49.876, *p* < .001), and there was a significant difference in consumer purchase intention for different categories of products; the presence or absence of a GM labeling had an impact on purchase intention (*F*(1, 596) = 5.416, *p* = .020) and consumers were influenced by the label information; the difference was that there was no interaction between the product category (soybean oil or cotton) and the label (whether containing GM or 68% GM) (*F*(1, 596) = 1.449, *p* = .229), that is, consumers’ responses to the label were similar in both the cotton scenario and the edible oil scenario. The impact of labels (qualitative or quantitative) does not change by product category. Detailed data are shown in Table 2 and Figure 3.

**Table 2.** Testing the main effects and interaction effects of experimental stimuli and situations.

Experimental stimuli	Source	SS (Type III)	df	MS	*F*	*p*
Non-GM Labels	scenario	31.360	1	31.360	19.430	<0.001
	labels	21.160	1	21.160	13.110	<0.001
	scenario×labels	22.090	1	22.090	13.687	<0.001
	error	639.140	396	1.614		
GM Labels	scenario	103.253	1	103.253	49.876	<0.001
	labels	11.213	1	11.213	5.416	0.020
	scenario×labels	3.000	1	3.000	1.449	0.229
	error	1233.850	596			

#### 4.2.2. Checking the Mediation Model

The results of the PROCESS Model 4 based on the overall sample context under non-GM label stimulation showed that perceived risk did not play a significant mediating role between label information and purchase intention (Effect = −0.0141, Boot *SE* = 0.021, 95%*CI* [−0.062, 0.024]). This result suggests that the “non-GM” label does not evoke a significant perception of product risk in individuals compared to the safety associations that the “GM” label may bring. This phenomenon may be explained from the perspective of consumers’ psychological preconceptions: despite public controversy over the safety of GM products, non-GM products are often regarded as “natural” and “safe” options and thus fail to trigger risk-related psychological responses. Given that the non-GM label does not significantly affect perceived risk and does not constitute a significant mediating pathway, the subsequent discussion will focus on the pathways through which the “GM” label affects the psychological mechanisms of consumers.

**Figure 4.**
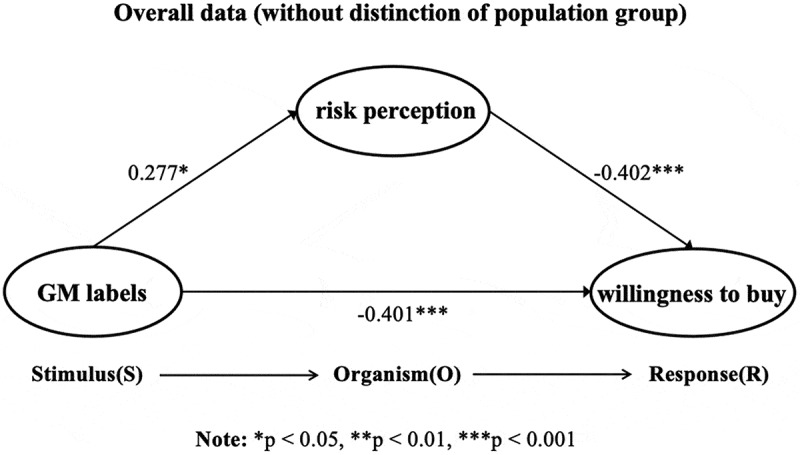
Results of the study hypothesis test.

Since previous between-subjects effect tests have shown that the interaction between “scenario × label” is not significantly affected by the GM label on different product categories, the context will not be broken down in the subsequent analysis. In the overall sample of GM labeling (*n* = 600), the study first conducted an independent sample t-test with different product labels (containing GM labels = 1/no special labels = 0) as independent variables and purchase desire as the dependent variable. The results show that in the overall situation without context discrimination (*n* = 600), the presence of the GM label (*M* = 4.298, *SD* = 1.468) significantly reduced the purchase intention (*t*(598 = 2.237, *p* = .026) of the subjects compared to the absence of this label (*M* = 4.995, *SD* = 1.458). The results of the mediating effect analysis (PROCESS Model 4) showed that the presence of the GM labeling significantly increased the risk perception of the subjects (*b* = 0.277, *SE* = 0.132, *t* = 2.092, *p* = .037, 95%*CI* [0.017, 0.536]), and risk perception significantly affected the participants’ desire to purchase the product (*b* = −0.402, *SE* = 0.037, *t* = −10.98, *p* < .001, 95%*CI* [−0.474, −0.330]). Further, the Bootstrap analysis results showed that risk perception negatively mediated the effect of GM labeling on the public’s desire to purchase the product (Effect = −0.111, Boot *SE* = 0.055, 95% *CI* [−0.222, −0.010]). Figure 4 shows the results of the hypothesis test for the study. H1, H2a, H2b, and H2c were validated in the entire sample.

**Figure 5.**
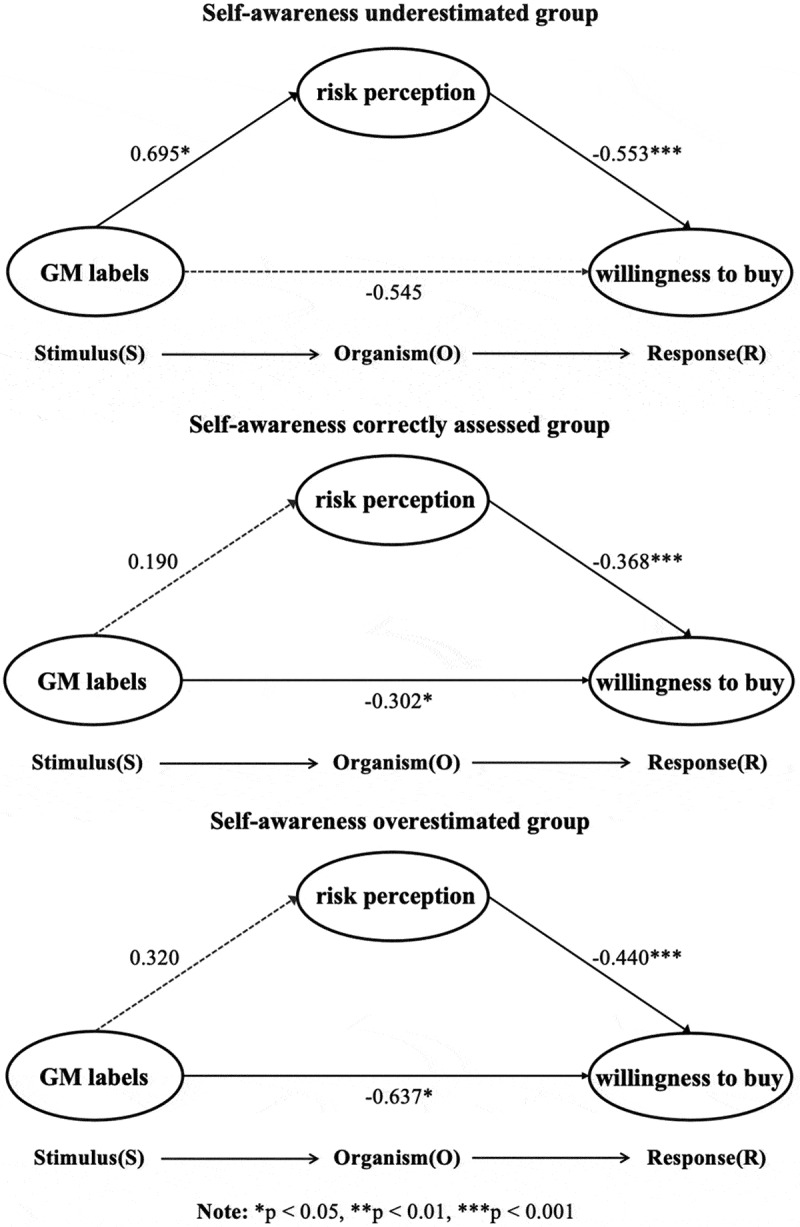
Pathways for different Dunning-Kruger effect groups.

To further explore the mediating role of perceived risk in different populations of the Dunning-Kruger effect, the subjects were divided into the self-perceived underestimation group (*n* = 80), correct assessment group (*n* = 381), and overestimation group (*n* = 139), and PROCESS Model 4 was used for mediating analysis. The results showed that in the self-perceived underestimation group, the GM labeling increased perceived risk (*b* = 0.695, *SE* = 0.301, *t* = 2.306, *p* = .024, 95%*CI* [0.095,1.295]), while perceived risk negatively affected purchase desire (*b* = −0.553, *SE* = 0.11, *t* = −4.854, *p* < .001, 95% *CI* [−0.780, −0.326]). Further analysis revealed that in the self-perceived underestimation group, perceived risk completely mediated the effect of the GM labeling on purchase intentions. The indirect effect was significant (Effect = −0.385, Boot *SE* = 0.167, 95%*CI* [−0.727,-0.086]), while the direct effect was no longer significant (*b* = −0.545, *SE* = 0.313, *t* = −1.739, *p* = .086, 95%*CI* [−1.170,0.079]), suggesting that perceived risk was the dominant factor in the group of self-perceived underestimation. Figure 5 shows the path effect in the self-perceived underestimation group.

In the correct assessment group, the mediating path was not significant (Effect = −0.070, Boot *SE* = 0.063, 95%*CI* [−0.051,0.198]). Although the direct path remained significant in this group (*b* = −0.302, *SE* = 0.142, *t* = −2.124, *p* = .034, 95%*CI* [−0.582,-0.023]), GM risk also negatively affected the desire to purchase (*b* = −0.368, *SE* = 0.044, *t* = −8.388, *p* < .001, 95% *CI* [−0.454, −0.282]. However, the effect of GM labeling did not affect perceived risk (*b* = 0.190, *SE* = 0.1664, *t* = −1.140, *p* = .255, 95%*CI* [−0.517,-0.138]). Figure 5 shows the path effect in the self-perceived correct assessment group.

Similarly, in the self-perceived overestimation group, the mediating path was not significant (Effect = −0.141, Boot *SE* = 0.1285, 95%*CI* [−0.110,0.402]). Although the GM label had a significant direct effect on desire to purchase (*b* = −0.637, *SE* = 0.283, *t* = −2.248, *p* = .026, 95%*CI* [−1.197,-0.077]), the impact of genetically modified risk on desire to purchase was also significant (*b* = −0.440, *SE* 0.082, *t* = −5.391, *p* < .001, 95% *CI* [−0.602, −0.279]. However, since the effect of GM labels on perceived risk was not significant (*b* = 0.320, *SE* = 0.295, *t* = 1.084, *p* = .280), the mediation did not hold. Figure 5 shows the path effect in the group with overestimated self-perception.

For both the self-perceived correct assessment group and the overestimation group, although perceived risk was significantly associated with purchase intention, it did not play a significant mediating role between GM labeling and purchase intention. Critically, GM labeling directly predicted purchase intention in these two groups, whereas no such direct effect emerged in the underestimation group. To summarize, distinct Dunning-Kruger effect groups (underestimating/correctly assessing/overestimating) generate differential pathway effects between GM labeling exposure and risk perception.

## 5. Discussion

Based on the exploration of the pathways through which GM labeling affects consumers’ purchase intentions, this study introduced the theoretical framework of the Dunning-Kruger effect and divided the subjects into underestimation, accurate assessment, and overestimation groups according to self-perceived assessment levels to examine the differences in the mediating effect of perceived risk. The study found that, firstly, both GM and non-GM labels significantly affect purchase intentions, but their effects pathways are different. The main effect of the GM label is context-independent and significantly reduces purchase intention (the interaction effect between the label and the product type is not significant) in both edible products (such as soybean oil) and non-edible products (such as cotton); non-GM labels, on the other hand, are context-dependent, interact significantly with product types, and have a significantly stronger positive promotional effect on edible products compared to non-edible products, indicating that the positive associations of “non-GM” labels need to be realized based on the context of health risks.

Secondly, product type significantly moderates risk perception. Consumers are more sensitive to issues surrounding foodstuffs. GM labeling is more likely to trigger physiological risk perception (such as health consequence associations) in the edible oil scenario, while risk perception is significantly weaker in the cotton scenario. This may be partly related to China’s long-standing naturalistic tendency toward food.^84^ Beyond cognitive factors, including risk perception, a large number of emotional factors influence consumers’ food choices,^50^ especially in the Chinese context.^35^ Due to long-term emotional dependence and a bias toward naturalism, Chinese people more tend to pursue “naturalism” at the food level, rejecting over-industrialized food and pursuing more organic food.^85^ Therefore, labeling something as non-GM implies a more naturalistic approach, which will more effectively enhance consumers’ willingness to purchase. However, this effect is not as prominent in other daily necessities such as textile products.

Finally, there are cognitive stratification differences in the mediating mechanism of perceived risk: in the self-underestimating group, perceived risk completely mediates the impact of labels on purchase intention (the label→risk perception→purchase intention path); in both the accurate assessment and overestimation groups, the label acted directly on purchase intentions, and the mediating effect was not significant, indicating that the judgment relied more on existing beliefs or attitudes (such as motivational reasoning) rather than risk information processing. This result suggests that it is necessary to understand the psychological framework of “cognitive misers” and “motivated reasoning” and its effect on the mechanism of information stimulation in cognitively confident individuals.^86,87^

Individuals with higher self-assessment usually have a higher level of confidence in their own knowledge and form a relatively stable belief system. Therefore, when faced with controversial information such as GM labeling, they are not easily affected by the “risk warning” mechanism triggered by the labels; instead, they are more likely to interpret the meaning of the label based on their existing positions, beliefs or values and make judgments that are in line with their own attitudes. This suggests that the interpretation path of labels as stimulus information is highly dependent on the consumer’s self-positioning and metacognitive ability. Therefore, other psychological pathways may exist to explain the relationship between labeling and purchase intention. This further underscores the multiplicity of psychological mechanisms underlying population heterogeneity.

The results validated the classic theory of GM labeling as “risk cues,”^74^ while expanding the boundaries of their effects through the Dunning-Kruger effect framework. From the perspective of the labeling effect itself, many studies have shown that “GM” tends to create a sense of “high risk,”^88^ and that labels activate risk associations rather than scientific knowledge during processing. This “label as risk” processing mechanism is consistent with the main effect found in this study, further supporting the heuristic cognitive pattern in which consumers tend to equate GM labeling with danger. However, previous studies have not further categorized the cognitive attributes of consumer groups. Starting from the Dunning-Kruger effect, this research provides results for further refinement of consumer groups and behaviors toward GM products with different cognitive characteristics. Based on confirming that GM labeling has a negative impact on consumers’ willingness to purchase GM products, this study further found that audiences with different self-perceptions respond differently to labels and purchase intentions. Self-underestimating groups are more likely to rely on external signals, such as product labels, to make judgments. They lack confidence in scientific issues and are thus more likely to be associated with risks by labels. In contrast, individuals who overestimate or accurately assess their own level of knowledge show higher cognitive stability, with labels having little or no influence on their behavioral choices through perceived risk, instead showing a direct effect, possibly due to their processing being driven more by existing experience.^89^ This provides a new perspective for the study of technology communication, emphasizing the need to differentiate between cognitive confidence levels and to develop differentiated strategies for low-cognitive groups and to manage the risk associations triggered by labels; for high self-rating groups, it may help to reconstruct their cognitive schemas to influence their decision-making logic.

This study uses the S-O-R model as a theoretical framework, embedding the cognitive assessment dimension as the individual characteristic variable, and proposing a path difference analysis method with “perceived risk” as the medium and “cognitive self-assessment” as the classification. Compared with previous studies on the effects of GM labeling, this path differentiation analysis not only refines the mechanism of the labeling effect but also emphasizes the importance of group differences to the overall efficiency of the technology communication model, suggesting that “communicating to whom” is more crucial than “communicating what.” Furthermore, in terms of the application of the Dunning-Kruger effect, most of the existing literature only focuses on verifying this effect^90^ and fails to explore its role on the effect of cognitive differences among different people. By introducing the framework of the Dunning-Kruger effect into the research scenario of risk communication, this study presents a possible path to explain why the same technological information produces different social effects in the population, enriches the bridging mechanism between cognitive assessment and information response, and promotes the transfer and application of psychological theory to the empirical scenario of communication studies. Furthermore, the findings of this study also help address existing research gaps by proposing a cross-contextual theoretical model that incorporates both group-level heterogeneity and product-related variations. By investigating these mechanisms in an understudied non-Western context, this work also yields empirical insights that question the universal applicability of Western-centric findings and informs the design of culturally and cognitively adapted labeling policies.

This study also provides empirical support and has a practical reference value for corporate marketing and the design of government labeling systems. However, it should be clarified firstly that there is a significant difference between the behavioral tendencies of online testing and real-life consumer behavior. Studies have shown that in ab online experimental context, people may manipulate their own negative perceptions, including risk.^91^ Therefore, caution is advised when applying the research conclusions. In the face of an increasingly complex technological information environment and differences in public perception, for GM governmental institutions, the policy design for GM product labels should explicitly abandon a uniform information strategy. Instead, regulators should develop a tiered or hierarchical information disclosure system that caters to varying public cognitive styles and information needs. This acknowledges that a single label triggers vastly different cognitive responses across segments of the population. And establishing formal mechanisms to continuously monitor and evaluate the real-world impact of labeling policies on consumer risk perception and behavior is also necessary for the GM policymakers, to further remains effective amidst a complex and evolving technological information environment. And enforcing clear and mandatory labeling requirements for GM produce can also enhance consumer trust and reduce their risks and skepticism.^92^ For the GM industry, companies must discard the notion of an “idealized consumer” in their marketing and communication strategies. Marketing efforts and label design should be adapted based on distinct cognitive profiles identified through research. This means developing multiple, clear messaging frameworks that resonate with different audience segments’ processing styles, and proactively providing clear, accessible information that addresses the specific concerns and information-processing habits of different consumer groups is also necessary. This involves moving beyond mere compliance with labeling regulations to building trust through tailored communication that helps various segments make informed judgments. And finally for the consumer groups, promoting targeted consumer education may be useful for the rational acceptance of GM labels. Developing and disseminating educational resources that are specifically tailored to address the knowledge gaps and concerns of different demographic and cognitive segments may further help to bridge the gap between perceived and actual risks. And consumer organizations need to play an effective intermediary role, and help the public make informed choices, relying on scientific evidence rather than opinion-based fears about GM products.^92^

## 6. Conclusion

GM labels are believed to have a significant impact on consumers’ GM consumption behavior, but its internal psychological mechanisms are unclear. This study focuses on the mediating mechanism of risk perception on the impact of GM labeling on consumption, as well as the potential impact of consumers’ Dunning-Kruger degree, based on the S-O-R model. Through an online experimental study, it was found that both GM and non-GM labels significantly affect purchase intentions, and this impact is significantly mediated by consumers’ risk perception. The mediating effect of risk perception is more pronounced in the self-perceived underestimation group (low Dunning-Kruger degree group). These results transcend the explanatory limitations of conventional linear models and provide empirical evidence for formulating differentiated GM labeling policies and optimizing risk communication strategies tailored to cognitive typologies.

## 7. Limitations

Although this study employed cluster path analysis and revealed some of the differential mechanisms of label information processing, there are some limitations. Firstly, although we used the G * Power method to test the adequacy of the samples, there may still be some sample bias within the online experimental method. Therefore, it is necessary to expand the scope of the sample and experimental environment in subsequent research, such as offline experiments. Secondly, the study did not explore whether non-GM labels have the effect of enhancing security. Although no significant difference was shown in risk perception, whether labels activate positive safety associations remains a psychological mechanism worthy of in-depth exploration, which can be interpreted and verified in the future from dimensions such as positive emotions or valence associations. It is necessary to further explore the impact of non-GM labels on consumers’ psychological cognition and the psychological pathways that influence their consumption behavior based on this study. Thirdly, the group division of the Dunning-Kruger effect is based on the difference method between self-assessment and knowledge score, lacking more rigorous testing standards, which is open to subjective interpretation in terms of measurement. Therefore, it is necessary to more accurately explore the direct impact of the Dunning-Kruger effect on consumer consumption behavior and its embedded psychological mechanisms from the perspective of behavioral and neuropsychological data. Finally, the research was conducted specifically in the Chinese context, where consumption habits vary from other countries, such as US and EU. Therefore, there may be certain difficulties in promoting the conclusions of this study on a global scale. More comprehensive comparative research is necessary in the future.

## Supplementary Material

Appendix.docx

## Appendix. The Questions About the Objective Knowledge of GMOs Used in the Questionnaires

**Table:**

Currently, the only genetically modified crop approved for commercial cultivation in China is cotton.	Yes	No
China allows the import of genetically modified food crop seeds for cultivation within its borders.		
Transgenic technology can reduce pesticide use.		
Transgenic technology can improve the nutritional content of crops.		
Currently, China has mandatory qualitative labeling for all products containing genetically modified materials on the market.		
As long as there are no genetically modified ingredients in the product, the “non genetically modified” label can be marked on the product.		
Currently, China implements a voluntary labeling system for agricultural genetically modified products.		
