# Supplementary material for: How labeling of genetically modified foods affects consumers’ purchase intentions: a multi-contextual analysis
Source: GM Crops Food. 2025 Oct 8;16(1):688–708. doi: 10.1080/21645698.2025.2572191 (PMC12520122; doi:10.1080/21645698.2025.2572191)
Supplement: Appendix.docx [file KGMC_A_2572191_SM1720.docx]

Appendix 1. The questions about the objective Knowledge of GMOs used in the questionaries

| Currently, the only genetically modified crop approved for commercial cultivation in China is cotton. | Yes | No |
| --- | --- | --- |
| China allows the import of genetically modified food crop seeds for cultivation within its borders. |  |  |
| Transgenic technology can reduce pesticide use. |  |  |
| Transgenic technology can improve the nutritional content of crops. |  |  |
| Currently, China has mandatory qualitative labelling for all products containing genetically modified materials on the market. |  |  |
| As long as there are no genetically modified ingredients in the product, the "non genetically modified" label can be marked on the product. |  |  |
| Currently, China implements a voluntary labeling system for agricultural genetically modified products. |  |  |
